# No evidence that selection for egg production persistency causes loss of bone quality in laying hens

**DOI:** 10.1186/s12711-021-00603-8

**Published:** 2021-02-04

**Authors:** Ian C. Dunn, Dirk-Jan De Koning, Heather A. McCormack, Robert H. Fleming, Peter W. Wilson, Björn Andersson, Matthias Schmutz, Cristina Benavides, Nazaret Dominguez-Gasca, Estefania Sanchez-Rodriguez, Alejandro B. Rodriguez-Navarro

**Affiliations:** 1 The Roslin Institute, University of Edinburgh, EH25 9RG Edinburgh, Scotland, UK; 2 Swedish University of Agricultural Sciences, 75651 Uppsala, Sweden; 3 Departamento de Mineralogía Y Petrologia, Universidad de Granada, 18002 Granada, Spain; 4 Lohmann Breeding, 7454 Cuxhaven, Germany

## Abstract

**Background:**

The physiological adaptations that have evolved for egg laying make hens susceptible to bone fractures and keel bone damage. In modern laying hen breeds, longer periods of egg laying could result in a greater risk of poor bone quality, and selection for increased egg production has frequently been stated to be a cause. However, the existing literature does not support this hypothesis. To test the hypothesis that egg production is associated with quality, breaking strength and density of bone, genetic correlations between these traits were estimated in White Leghorn and Rhode Island Red breeds. Genetic correlations of cortical and medullary bone material chemical properties with bone quality were also estimated, in order to identify methods to improve bone quality with appropriately targeted measurement of key traits.

**Results:**

Estimates of heritability for bone quality traits were moderate (0.19–0.59) for both White Leghorn and Rhode Island Red breeds, except for the keel bone trait, which had a heritability estimate equal to zero. There was no evidence for genetic or phenotypic relationships between post-peak egg production and bone quality. In the White Leghorn breed, the estimate of the genetic correlation between pre-peak production/age at first egg and bone quality was significant and negative (− 0.7 to − 0.4). Estimates of heritability of thermogravimetric measurements of tibial medullary bone mineralisation were significant (0.18–0.41), as were estimates of their genetic correlations with tibia breaking strength and density (0.6–0.9).

**Conclusions:**

The low genetic correlation of post-peak egg production with bone quality suggests that selection for increased persistency of egg production may not adversely affect bone quality. Onset of puberty and mineralisation of the medullary bone, which is a specialised adaptation for egg laying, were identified as important factors associated with the quality of the skeleton later during egg production. These are traits for which genetic, as well as environmental and management factors can positively impact the overall quality of the skeleton of laying hens.

## Background

Bone fractures are a challenge for laying hens [1], since physiological adaptations for egg laying make them susceptible to osteoporosis [2]. There is also a welfare paradox in laying hens, i.e. cage-free production, which allows greater movement and mechanical loading of the bone, is consistently associated with increased bone quality but, at the same time, these alternative systems are associated with a greater incidence of bone damage [3], often featuring the keel [4]. There is also a fear that the increasingly persistent laying hen, which has great benefits for sustainability but leads to a longer period of production, may be associated with greater risk of poor bone quality in end-of-lay hens [5]. In this context, it is frequently stated, including by ourselves, that poor bone quality is caused by intense selection for increased egg production [5–8]. However, the literature on these topics does not support this hypothesis. While the physiology of laying eggs, as opposed to not laying eggs, can be related to potential issues of poor bone quality, it is not clear that the phenotypic relationship between number of eggs laid and bone quality is actually negative [9].

The answer to the question how egg production affects bone quality seems simple, because chickens that do not lay eggs, either because they are males or females that have been prevented from laying eggs, have virtually no bone problems [10, 11]. It seems likely that the absence of oocytes and the resulting lack of female sex steroids are the cause of this difference. Although we cannot rule out this possibility, genetic effects of sexual dimorphism directly through growth [12] or its underlying endocrinology [13] may contribute to the differences in bone fracture susceptibility between sexes.

The large number of eggs that the modern hybrid layer produces is an attractive explanation for its deficiencies in bone quality, perhaps because the concept that we are pushing animals to their biological limits is a popular one [14]. However, several observations have led us to question this assumption. In particular, it is evident that hens with symptoms of poor bone quality, especially the keel, have been observed for quite a long time. For example, Darwin’s 1868 book on ‘The variation of animals and plants under domestication’ [15] reports issues regarding bone quality in poultry, especially of the keel: ‘This bone is generally so much deformed that it is scarcely possible to compare its shape strictly in the several breeds’. It was also reported that up to 88% of chickens examined had a deformed sternum and that most of these were males [15]. Archaeological evidence for the breakage and healing of a bone during the life of a hen in the seventh/eighth century AD has been documented [16]. More contemporaneously, at the start of the application of modern genetic selection, evidence of keel bone deformities has been reported [17]. A comparison of related lines that had been selected more or less intensively for egg production, provided limited evidence that the more intensely selected lines had worse bone quality [18, 19]. In another comparison between two high egg producing breeds, the breed with the highest egg production actually had better bone quality, as measured by keel bone deviation [20]. However, when egg mass instead of egg number was considered, the more productive breed was the breed with the poorer quality bone [20]. Finally, Fleming et al. [21] reported that selection for bone quality was possible without reducing egg production, which indicates that the two factors are not necessarily closely genetically linked.

What is clear is that improving bone quality in laying hens, especially the keel, is an important welfare breeding goal [22, 23]. To tackle this objective effectively, it is necessary to understand what factors influence bone quality. In this study, our first aim was to estimate the genetic correlation between egg production and bone quality. Our second objective was to determine which, if any, measurements of the chemical constituents of bone correlate genetically with the mechanical or density properties of avian bone.

## Methods

### Animals

#### White Leghorn breed

The White leghorn (WL) chickens were from two (WLa and WLb) later generations of the line that was investigated in previous studies on bone quality [24, 25] and that is used to produce LSL hybrid layers (Lohmann Tierzucht GmbH). WLa chickens (n = 933) were from the same population as used in our previous GWAS study [26] and bone samples were collected between 62 and 64 weeks of age from three hatches. WLb chickens (n = 930) have not been studied before and samples were collected at 53 weeks of age from two hatches. This is an earlier age than used in previous studies due to restrictions on housing. Hens were housed with a companion hen in cages that were equipped with a perch. Egg colour allowed individual egg recording. Although the WLa hens were older and had less information than the WLb hens, their data were used to confirm results from the more contemporary WLb hens.

#### Rhode Island Red breed

The Rhode Island Red (RIR) chickens (n = 925) were from a later generation of the line that was investigated in previous studies on egg quality [27, 28] and that is used to produce Lohmann Brown commercial layers (Lohmann Tierzucht GmbH, Germany). Samples were collected at 68 weeks of age from three hatches. Hens were housed with a companion hen in cages that were equipped with a perch. Again, egg colour allowed individual egg recording.

### Bones sampled

In this study, we examined the humerus and tibia, which are both part of the appendicular skeleton, and the keel or sternum, which is part of the axial skeleton. The humerus and tibia have more or less load bearing roles with a cortical structure and, in mature female animals, they have varying amounts of medullary bone in the central cavity. The keel, at least in flying or swimming birds, has load applied from the action of the pectoral muscles which are attached to the keel structure and are necessary for wing flapping. The keel also contains some medullary bone which can be found between the lateral plates that form the structure in birds.

### Body mass and egg production recording

Body mass was recorded at 26, 53 (cull age), and 68 weeks of age for WLa, WLb, and RIR hens, respectively. For WLb and RIR, early egg number, which is defined as the number of eggs produced in the first two recording periods, was analysed separately. Early egg number is related to age at first egg (AFE) because hens that have an earlier AFE have a larger egg number in that period. Egg number in the remaining period represented post-peak production and is referred to as egg number. For both lines, egg recording started at 19 weeks of age and therefore the first two periods represent the 19–26 weeks ages. Egg mass is the average mass of eggs collected at 26 and 38 weeks of age for WLb and 26, 36 and 48 weeks of age for RIR. Egg breaking strength is an average value of two eggs at 45 weeks of age for WLb and RIR.

Although body mass has a role on bone quality parameters, we did not correct or fit body mass in the analyses presented here. We believe that it is easier to detect relevant interactions between traits if the data are not adjusted. However, the effect of fitting body mass for the key effects on bone breaking strength was included in the results.

### Bone material measurements

#### Breaking strength

The biomechanical properties of the tibia and humerus bones were evaluated by breaking strength, which was determined by a three-point bending test using a materials testing machine (JJ Lloyd LRX50, Sussex, UK), as previously described [29]. Data were available for both bones of WLb and RIR hens but only for the tibia of WLa hens.

#### Radiographic denity

Radiographic density data were available for the WLb and RIR hens. Whole tibia, humeri, and sterna (keel bones) were radiographed in a Faxitron 43855D soft X ray apparatus fitted with an NTB EZ240 digital x-ray scanner (NTB GmbH, Germany). Voltage of exposure was adjusted for each bone type and age of bird. For calibration purposes, each exposure included a 16-step aluminium step wedge, with 0.25 mm increments. Images were acquired using the IX-Pect acquisition and imaging software supplied with the scanner.

The radiographic density of the bones was determined using the software package ImageJ 1.32 (http://rsb.info.nih.gov/ij/). Each bone was automatically delineated from the background and the mean radiographic density (pre-calibrated in mm of aluminium equivalent) of the whole bone was measured. The proportion of medullary and cortical bone type was calculated directly from the X-ray by delineation [30].

#### Bone mineral chemistry and structure

The chemical composition, bone mineral crystallinity, and organization of (cortical and medullary) bone tissue from the mid shaft tibia were measured on WLb and RIR hens using infrared (IR) spectroscopy, thermogravimetry (TGA), and X-ray diffraction (XRD), as described previously [31, 32].

IR spectra of cortical and medullary bone samples provided the following compositional parameters that are based on major absorption peaks: (1) the PO_4_/AmideI ratio, which measures the degree of mineralization in bone; bone mineral is made of carbonate apatite (calcium phosphate) and the main peak at 900–1200 cm^−1^ for PO_4_ was used to quantify the amount of mineral in the bone; the bone organic matrix is made of collagen and non-collagen proteins and the main peak of proteins at 1640 cm^−1^ for Amide I was used to quantify the amount of organic matrix in the bone; (2) the CO_3_1415/AmideI ratio, which estimates the amount of carbonate (a mineral component) relative to the organic matrix component in bone; the main IR band at 1415 cm^−1^ for carbonate was used to quantify the amount of carbonate; different amounts of carbonate substitution in the apatite result in altered properties of the bone mineral (dissolution); (3) MinCO_3_1415, which measures the total amount of carbonate in bone mineral and is calculated as the ratio of the main carbonate band at 1415 cm^−1^ to the main phosphate band at 900–1200 cm^−1^; this measurement is indicative of bone mineral maturation and bone remodeling rate (decreases with bone mineral maturation and bone tissue age); and (4) MinCO_3_870, which is the amount of carbonate substituted in the mineral and was calculated as the ratio of the carbonate band at 870 cm^−1^ to the main phosphate band at 900–1200 cm^−1^; this measurement is also indicative of bone mineral maturation.

Thermogravimetric analysis was used to estimate the percentages of water, organic matter, mineral (phosphate + carbonate) in cortical and medullary bone samples. Powdered bones were treated at 200, 600, and 800 °C in a RWF 1100 furnace (Carbolite, UK) for 1 h and weighed to determine the weight fraction (0–100) of water (H_2_O%), organic matrix (Organic%), and mineral (Mineral%). The amount of carbonate (CO_3_%) in the mineral part is determined as the weight loss in the 600–800 °C range, the remaining weight at 800 °C is phosphate (PO_4_%) in the mineral. Note that although bone mineral is composed of carbonate apatite, Ca_5_(PO_4_)_3_(CO_3_), the calcium phosphate part is referred to as PO_4_ and carbonate as CO_3_ for consistency with IR spectroscopy nomenclature. From these measurements, the degree of mineralization (PO_4_/Organic) and the relative content of carbonate in the mineral (CO_3_/Mineral) were calculated, which are comparable to similar measurements by IR spectrometry (PO_4_/AmideI and MinCO_3_1415).

Bone mineral crystallinity and organization (crystal orientation) were quantified from the following XRD parameters: (1) FWHM_002 (deg), which is the width of the 002 apatite peak (FWHM) and represents an estimate of the length of apatite crystals, which decreases with bone mineral crystallinity; (2) FWHM_310 (deg), which is the width of the 310 apatite peak (FWHM) and represents an estimate of the width of apatite crystals, which also decreases with bone mineral crystallinity; (3) AS_002, which represents the angular scattering of apatite crystals within bone mineral; and (4) oriented fraction, which represents the relative amount of apatite crystals that are preferentially oriented in the mineral. Note that the latter two parameters were calculated only for cortical bone since the mineral is randomly oriented in medullary bone.

## Statistical methods

Analyses were carried out by using mixed linear models in the ASReml 4 software (VSN International). A bivariate model with the following terms was fitted for each breed:

$$\left[ {\begin{array}{*{20}c} {{\mathbf{y}}_{1} } \\ {{\mathbf{y}}_{2} } \\ \end{array} } \right] = \left[ {\begin{array}{*{20}c} {1\mu_{1} } \\ {1\mu_{2} } \\ \end{array} } \right] + \left[ {\begin{array}{*{20}c} {{\mathbf{X}}_{{1}} } & {\mathbf{0}} \\ {\mathbf{0}} & {{\mathbf{X}}_{{2}} } \\ \end{array} } \right]\left[ {\begin{array}{*{20}c} {{{\varvec{\upbeta}}}_{1} } \\ {{{\varvec{\upbeta}}}_{2} } \\ \end{array} } \right] + \left[ {\begin{array}{*{20}c} {{\mathbf{Z}}_{{1}} } & {\mathbf{0}} \\ {\mathbf{0}} & {{\mathbf{Z}}_{{2}} } \\ \end{array} } \right]\left[ {\begin{array}{*{20}c} {{\mathbf{u}}_{1} } \\ {{\mathbf{u}}_{2} } \\ \end{array} } \right] + \left[ {\begin{array}{*{20}c} {{\mathbf{e}}_{1} } \\ {{\mathbf{e}}_{2} } \\ \end{array} } \right],$$

where ${\mathbf{y}}_{1}$ and ${\mathbf{y}}_{2}$ are column vectors of two traits (where subscripts 1 and 2 are taken from the traits described in the sections ‘Bone material properties’ and ‘Production traits’); ${\mu }_{1}$ and ${\mu }_{2}$ are their respective means, with **1** a column vector of 1s; ${{\varvec{\upbeta}}}_{1}$ and ${{\varvec{\upbeta}}}_{2}$ are additional fixed effects for hatches, with ${\mathbf{X}}_{1}$ and ${\mathbf{X}}_{2}$ their respective design matrices; ${\mathbf{u}}_{1}$ and ${\mathbf{u}}_{2}$ are vectors of animal breeding values for the traits, with ${\mathbf{Z}}_{1}$ and ${\mathbf{Z}}_{2}$ their respective design matrices; and ${\mathbf{e}}_{1}$ and ${\mathbf{e}}_{2}$ are vectors of residual errors. In addition to the mean of the trait, the fixed effects were 2 degrees of freedom (df) per trait for line WLa, 1 df per trait for line WLb, and 3 df per trait for RIR. Breeding values $({{\mathbf{u}}_{1}^{\mathrm{T}}, {\mathbf{u}}_{2}^{\mathrm{T}})}^{\mathrm{T}}$ were assumed distributed as ${\text{MVN}}\,\left( {{\mathbf{0}},{\mathbf{A}} \otimes {\mathbf{U}}} \right)$, where $\mathbf{A}$ is the numerator relationship matrix generated from the pedigree (the depth of the pedigree was 2 generations for all lines), and $\mathbf{U}$ is a $2\times 2$ matrix of genetic (co)variances; for a $t$ trait model, $\mathbf{U}$ was $t\times t$. For elements of $\mathbf{U}$, the genetic variance for trait $i$ is denoted ${\upsigma }_{\mathrm{A},i}^{2}$ and the covariance between traits $i$ and $j$ is ${\mathrm{r}}_{\mathrm{A},ij}{\upsigma }_{\mathrm{A},i}{\upsigma }_{\mathrm{A},j}$, where ${\mathrm{r}}_{\mathrm{A},ij}$ is the additive genetic correlation between the traits. Residuals $({{\mathbf{e}}_{1}^{\mathrm{T}}, {\mathbf{e}}_{2}^{\mathrm{T}})}^{\mathrm{T}}$ were assumed distributed as ${\text{MVN}}\,\left( {{\mathbf{0}},{\mathbf{I}} \otimes {\mathbf{V}}} \right)$, where $\mathbf{I}$ is the identity matrix and $\mathbf{V}$ is a $2\times 2$ matrix (or $t\times t$ for $t$ traits). For elements of $\mathbf{V}$, the residual variance for trait $i$ is denoted ${\upsigma }_{\mathrm{E},i}^{2}$ and the covariance between traits $i$ and $j$ is ${\mathrm{r}}_{\mathrm{E},ij}{\upsigma }_{\mathrm{E},i}{\upsigma }_{\mathrm{E},j}$, where ${\mathrm{r}}_{\mathrm{E},ij}$ is the environmental correlation between traits. For trait $i,$ the phenotypic variance was calculated as ${\upsigma }_{\mathrm{P},i}^{2}={\upsigma }_{\mathrm{A},i}^{2}+{\upsigma }_{\mathrm{E},i}^{2}$ and heritability was calculated as ${h}_{i}^{2}={\upsigma }_{\mathrm{A},i}^{2}/{\upsigma }_{\mathrm{P},i}^{2}$.

## Results

### Bone quality and production traits

#### Summary statistics

The ‘early egg’ production data (Table 1) demonstrate that egg production was just below 50% for the first two 4-week periods. At the start of the recording period none of the hens were laying eggs but by the end of the period all hens were laying eggs. Flock production reached 50% in the middle of the 8-week period.

**Table 1:** Summary statistics for egg production, body mass, and bone traits for the White leghorn a and b hens at 63 and 53 weeks of age, respectively, and Rhode Island Red hens at 68 weeks of age

Trait	Number of individuals	Mean ± sem
White Leghorn population a		
Cull body mass (g)	933	1620 ± 5
Tibia breaking strain (N)	933	207.9 ± 1.6
Total egg number	933	240.8 ± 0.4
White Leghorn population b		
Cull body mass (g)	969	1645 ± 4
Early egg number	972	24.33 ± 0.20
Late egg number	972	151.8 ± 0.2
Average egg mass (g)	972	58.5 ± 0.1
Egg breaking strain 45 weeks (N)	972	42.5 ± 0.2
Humerus breaking strain (N)	958	178.1 ± 1.0
Humerus density (mm Al equivalent)	955	1.092 ± 0.003
Keel density (mm Al equivalent)	958	0.623 ± 0.003
Tibia breaking strain (N)	969	215.6 ± 1.3
Tibia density (mm Al equivalent)	970	2.056 ± 0.005
Rhode Island Red population		
Cull body mass (g)	918	1907 ± 6
Early egg number	925	29.9 ± 0.3
Average egg mass (g)	972	63.3 ± 0.1
Egg breaking strain 45 weeks (N)	972	54.2 ± 0.3
Late egg number	925	245.1 ± 0.43
Humerus breaking strain (N)	885	160.8 ± 1.7
Humerus density (mm Al equivalent)	886	1.50 ± 0.01
Keel density (mm Al equivalent)	884	0.829 ± 0.003
Tibia breaking strain (N)	916	230.4 ± 1.8
Tibia density (mm Al equivalent)	916	2.370 ± 0.009

On average, the RIR hens were heavier than the WLa and WLb hens (Table 1), which may explain in part their higher tibia breaking strength due to increased loading. However, this was not the case for the humerus breaking strength (Table 1).

#### Genetic parameters

For the WLa hens, a limited amount of data was available (see Table 1). The heritability estimates for tibia breaking strength and total egg production were 0.69 ± 0.08 and 0.23 ± 0.07, respectively, and there was no significant genetic correlation between these two traits (0.02 ± 0.16).

For the WLb and RIR populations, more data were available (Table 2). Heritability estimates for tibia and humerus bone quality traits ranged from 0.19 ± 0.07 to 0.48 ± 0.08 for WLb and from 0.36 ± 0.08 to 0.59 ± 0.09 for RIR (Table 2). In general, heritability estimates were higher for tibia measurements than for humerus measurements, but not exclusively. The heritability estimate for keel bone density was near zero. The heritability estimate was higher for early egg production than for late egg production, which occurred post-peak, but both were significantly higher than zero (Table 2).

**Table 2:** Estimates of heritabilities (diagonal), genetic correlations (below the diagonal), and environmental correlations (above the diagonal), with standard errors, between egg production, body mass, and bone traits for the White Leghorn b (WLb) and Rhode Island Red (RIR) populations

	Humerus breaking strain	Humerus density	Tibia breaking strain	Tibia density	Keel density	Early egg (1st two periods)	Late egg number	Egg breaking strain	Average egg mass	Cull body mass
White Leghorn b (WLb)										
Humerus breaking strain (N)	**0.30 ± 0.07**	**0.37 ± 0.05**	**0.30 ± 0.06**	0.06 ± 0.08	− 0.07 ± 0.08	0.03 ± 0.08	0.12 ± 0.06	0.04 ± 0.06	0.07 ± 0.08	0.07 ± 0.09
Humerus density (mm Al equiv)	**0.68 ± 0.15**	**0.19 ± 0.06**	**0.25 ± 0.06**	**0.18 ± 0.07**	0.08 ± 0.08	0.09 ± 0.08	− 0.07 ± 0.06	0.04 ± 0.06	0.02 ± 0.07	0.07 ± 0.07
Tibia breaking strain (N)	**0.76 ± 0.14**	**0.43 ± 0.20**	**0.24 ± 0.07**	**0.47 ± 0.06**	0.08 ± 0.05	0.14 ± 0.07	− 0.02 ± 0.06	0.00 ± 0.06	0.00 ± 0.08	**0.31 ± 0.07**
Tibia density (mm Al equiv)	**0.50 ± 0.15**	**0.60 ± 0.14**	**0.84 ± 0.07**	**0.48 ± 0.08**	0.17 ± 0.11	0.13 ± 0.09	− 0.00 ± 0.067	− 0.05 ± 0.07	− 0.05 ± 0.09	**0.24 ± 0.08**
Keel density (mm Al equiv)	0.54 ± 0.31	**0.83 ± 0.31**	0.74 ± 0.39	0.58 ± 0.29	0.03 ± 0.04	0.06 ± 0.06	0.01 ± 0.06	0.07 ± 0.05	− 0.02 ± 0.06	− 0.02 ± 0.06
Early egg number (1st two periods)	− **0.44 ± 0.18**	− **0.51 ± 0.20**	− **0.73 ± 0.15**	− **0.60 ± 0.13**	− 0.68 ± 0.59	**0.34 ± 0.08**	− **0.27 ± 0.06**	− 0.07 ± 0.07	− 0.12 ± 0.09	0.14 ± 0.08
Later egg number	− 0.02 ± 0.24	0.36 ± 0.25	0.25 ± 0.25	0.24 ± 0.21	− 0.10 ± 0.69	− **0.71 ± 0.16**	**0.16 ± 0.06**	0.01 ± 0.06	0.06 ± 0.07	0.00 ± 0.07
Egg breaking strain 45 weeks (N)	− 0.01 ± 0.23	− 0.11 ± 0.26	0.10 ± 0.24	0.01 ± 0.21	− 0.81 ± 0.88	0.09 ± 0.23	− 0.06 ± 0.28	**0.17 ± 0.06**	− 0.03 ± 0.07	**0.18 ± 0.07**
Average egg mass (g)	0.23 ± 0.16	0.31 ± 0.19	0.08 ± 0.18	0.28 ± 0.14	0.11 ± 0.45	− 0.08 ± 0.16	− 0.08 ± 0.21	− 0.35 ± 0.19	**0.45 ± 0.08**	0.07 ± 0.09
Cull Body Mass (g)	**0.53 ± 0.09**	**0.47 ± 0.18**	0.20 ± 0.18	**0.38 ± 0.14**	0.49 ± 0.66	− **0.64 ± 0.13**	0.20 ± 0.17	− **0.36 ± 0.17**	**0.55 ± 0.09**	**0.36 ± 0.08**
Rhode Island Red (RIR)										
Humerus breaking strain (N)	**0.47 ± 0.08**	**0.52 ± 0.06**	− 0.00 ± 0.11	− 0.03 ± 0.12	− 0.03 ± 0.07	− **0.26 ± 0.08**	0.01 ± 0.07	− 0.01 ± 0.07	0.21 ± 0.11	0.08 ± 0.09
Humerus density (mm Al equiv)	**0.62 ± 0.10**	**0.36 ± 0.08**	− 0.07 ± 0.10	− 0.05 ± 0.11	0.12 ± 0.06	− 0.08 ± 0.08	0.02 ± 0.06	0.11 ± 0.07	0.07 ± 0.10	− 0.05 ± 0.05
Tibia breaking strain (N)	**0.81 ± 0.07**	**0.71 ± 0.10**	**0.51 ± 0.08**	**0.53 ± 0.08**	**0.20 ± 0.07**	0.06 ± 0.09	− **0.18 ± 0.07**	0.11 ± 0.08	0.20 ± 0.12	**0.13 ± 0.06**
Tibia density (mm Al equiv)	**0.52 ± 0.11**	**0.65 ± 0.11**	**0.83 ± 0.05**	**0.59 ± 0.09**	**0.51 ± 0.06**	0.05 ± 0.10	− 0.16 ± 0.08	0.06 ± 0.08	**0.35 ± 0.13**	**0.22 ± 0.07**
Keel density (mm Al equiv)	**0.50 ± 0.25**	0.42 ± 0.26	**0.72 ± 0.19**	0.44 ± 0.23	0.08 ± 0.05	0.04 ± 0.06	0.05 ± 0.05	0.13 ± 0.06	0.13 ± 0.08	0.06 ± 0.04
Early egg number (1st two periods)	− 0.15 ± 0.14	− 0.07 ± 0.17	− 0.25 ± 0.14	− 0.20 ± 0.14	− 0.15 ± 0.27	**0.40 ± 0.08**	− 0.05 ± 0.06	0.08 ± 0.06	− 0.19 ± 0.10	0.04 ± 0.06
Later egg number	0.02 ± 0.22	− 0.35 ± 0.24	− 0.08 ± 0.21	− 0.05 ± 0.22	− 0.38 ± 0.38	0.15 ± 0.23	**0.11 ± 0.05**	0.06 ± 0.05	− 0.07 ± 0.08	0.07 ± 0.04
Egg breaking strain 45 weeks (N)	0.16 ± 0.21	0.06 ± 0.24	− 0.35 ± 0.19	− 0.26 ± 0.20	− **0.78 ± 0.30**	− 0.05 ± 0.21	0.11 ± 0.32	**0.14 ± 0.06**	− 0.02 ± 0.08	0.09 ± 0.05
Average egg mass (g)	− 0.21 ± 0.14	− 0.06 ± 0.10	− **0.27 ± 0.13**	− 0.21 ± 0.13	− 0.03 ± 0.26	− **0.33 ± 0.13**	− 0.12 ± 0.22	− 0.13 ± 0.20	**0.58 ± 0.08**	**0.26 ± 0.07**
Cull body mass (g)	0.17 ± 0.16	**0.25 ± 0.11**	**0.25 ± 0.09**	**0.21 ± 0.10**	0.29 ± 0.19	− 0.02 ± 0.10	0.10 ± 0.16	− **0.32 ± 0.14**	0.03 ± 0.09	**0.44 ± 0.04**

Figures in bold are considered significant

Estimates of genetic correlations between late egg number and bone quality were not significantly different from zero (Table 2); in some cases, the values were quite high but with large standard errors. The sign of the correlation estimates was predominately positive for WLb hens and negative for RIR hens (Table 2). For example, the estimates of the genetic correlation between late egg production and tibia bone density were 0.24 ± 0.21 and − 0.05 ± 0.22 for WL and RIR, respectively. However, for WLb, the estimates of the genetic correlation between bone quality traits and early egg number were significantly negative, e.g. for tibia density and tibia breaking strength − 0.60 ± 0.13 and − 0.73 ± 0.15, respectively. No similar relationship with early egg production was found for RIR hens being 0.25 ± 0.25 and 0.24 ± 0.21, for tibia density and tibia breaking strength, respectively (Table 2).

Estimates of genetic correlations were also negative between early egg number and body mass in the WLb hens (− 0.64 ± 0.13). In the WLb hens, body mass at cull also had a significant positive genetic correlation estimate with several bone quality traits and egg mass, as expected, and a negative genetic correlation estimate with egg breaking strength at 45 weeks of age (Table 2). Except for the correlation of cull body mass with early egg production and egg mass, correlation estimates were similar in the RIR population (Table 2). In the WLb population, number of eggs laid in the early period and number of eggs laid post-peak, were genetically negatively correlated, which was not found in the RIR population where it was positive with a large error.

In terms of the environmental correlations, significant positive correlations between bone quality traits were observed in both the WLb and RIR populations, particularly for different traits on a given bone type, and a positive correlation of cull mass with tibia bone quality traits was found (Table 2). We did not account for body mass in the analysis presented (Table 2). Including cull mass in the model for tibia breaking strength did not alter the estimate of heritability, but the estimate of the genetic correlation between tibia breaking strength residual and early egg in WLb changed from − 0.73 ± 0.15 to − 0.49 ± 0.16 and remained significantly different from zero.

Estimates of phenotypic correlations between different bone quality traits and between bone quality traits and cull mass were positive and significantly different from zero (Table 3). Estimates of phenotypic correlations between bone quality and early egg production were largely significant and negative in the WLb line but not significant in the RIR line (Table 3).

**Table 3:** Estimates of phenotypic correlations, with 95% confidence intervals in square brackets, between egg production, body mass, and bone traits for the White Leghorn b (WLb) and Rhode Island Red (RIR) population

	Humerus density	Tibia breaking strain	Tibia density	Keel density	Early egg (1st two periods)	Late egg number	Egg breaking strain	Average egg mass	Cull body mass
White Leghorn (WLb)									
Humerus breaking strain (N)	**0.44 [0.39 0.49]**	**0.40 [0.34 0.45]**	**0.22 [0.16 0.28]**	0.06 [− 0.01 0.12]	− **0.17 [**− **0.23 **− **0.11]**	**0.11 [0.04 0.17]**	0.04 [− 0.02 0.11]	**0.11 [0.05 0.17]**	**0.23 [0.17 0.29]**
Humerus density (mm Al equiv)		**0.26 [0.20 0.32]**	**0.32 [0.26 0.38]**	**0.24 [0.18 0.30]**	− **0.22 [**− **0.28 **− **0.16]**	0.04 [− 0.28 − 0.16]	0.03 [− 0.03 0.09]	**0.07 [0.01 0.14]**	**0.14 [0.08 0.21]**
Tibia breaking strain (N)			**0.56 [0.52 0.60]**	**0.14 [0.08 0.20]**	− 0.04 [− 0.11 0.02]	0.02 [− 0.04 0.08]	0.02 [− 0.04 0.08]	0.03 [− 0.03 0.09]	**0.33 [0.27 0.38]**
Tibia density (mm Al equiv)				**0.29 [0.23 0.34]**	− **0.18 [**− **0.24 **− **0.12]**	**0.07 [0.01 0.14]**	− 0.02 [− 0.09 0.04]	**0.08 [0.02 0.14]**	**0.31 0.25 0.36]**
Keel density (mm Al equiv)					− 0.12 [− 0.19 − 0.06]	0.02 [− 0.04 0.08]	0.04 [− 0.02 0.10]	− 0.02 [− 0.08 0.05]	0.01 [− 0.05 0.07]
Early egg number (1st 2 periods)						− **0.35 [**− **040 **− **0.29]**	− **0.10 [**− **0.16 **− **0.03]**	0.00 [− 0.05 0.07]	− 0.00 [− 0.06 0.06]
Later egg number							0.01 [− 0.06 0.07]	0.00 [− 0.06 0.06]	0.04 [− 0.03 0.10]
Egg breaking strain 45 weeks (N)								− **0.12 [**− **0.19 **− **0.06]**	− 0.00 [− 0.07 0.06]
Average egg mass (g)									**0.31 [0.25 0.37]**
Rhode Island Red (RIR)									
Humerus breaking strain (N)	**0.56 [0.51 0.60]**	**0.41 [0.35 0.46]**	**0.32 [0.26 0.38]**	**0.11 [0.05 0.18]**	0.03 [− 0.04 0.09]	− 0.06 [− 0.13 0.00]	− 0.05 [− 0.11 0.02]	− 0.02 [− 0.09 0.04]	**0.11 [0.04 0.17]**
Humerus density (mm Al equiv)		**0.28 [0.21 0.34]**	**0.29 [0.23 0.35]**	**0.19 [0.13 0.25]**	− 0.00 [− 0.07 0.07]	− **0.08 [**− **0.14 **− **0.01]**	**0.07 [0.01 0.14]**	0.01 [− 0.06 0.07]	**0.08 [0.01 0.15]**
Tibia breaking strain (N)			**0.69 [0.65 0.72]**	**0.30 [0.24 0.35]**	− 0.03 [− 0.10 0.03]	− **0.14 [**− **0.21 **− **0.08]**	− 0.05 [− 0.12 0.01]	− **0.07 [**− **0.13 **− **0.01]**	**0.25 [0.18 0.31]**
Tibia density (mm Al equiv)				**0.49 [0.43 0.56]**	0.03 [− 0.04 0.09]	− **0.14 [**− **0.21 **− **0.08]**	− 0.07 [− 0.04 0.09]	0.02 [− 0.04 0.09]	**0.27 [0.21 0.33]**
Keel density (mm Al equiv)					0.04 [− 0.03 0.10]	− **0.10 [**− **0.16 **− **0.03]**	0.00 [− 0.06 0.07]	**0.07 [0.00 0.14]**	0.13 [0.07 0.20]
Early egg number (1st 2 periods)						− **0.10 [**− **0.16 **− **0.03]**	− 0.03 [− 0.09 0.04]	− **0.23 [**− **0.29 **− **0.17]**	− 0.00 [− 0.07 0.06]
Later egg number							**0.09 [0.02 0.15]**	− 0.07 [− 0.14 − 0.01]	**0.09 [0.03 0.16]**
Egg breaking strain 45 weeks (N)								− 0.04 [− 0.10 0.03]	− 0.01 [− 0.08 0.05]
Average egg mass (g)									**0.16 [0.10 0.22]**

Figures in bold are significantly different from zero (P < 0.05)

### Bone material chemical properties

#### Summary statistics

We did not perform statistical comparisons for mineral composition of the medullary and cortical components of the mid shaft tibia (Table 4) between breeds or between bone type data because of differences in bird age between the breeds and sampling periods. That said, there were clear differences between medullary and cortical bone types, with a greater mineral content in the cortical bone, as reflected by the PO_4_/organic ratio (Table 4). There was also a putative breed difference for carbonate content, which was higher in WLa than in RIR hens, as reflected by the CO_3_/mineral ratio values (Table 4). We also observed putative breed differences in the mineral organization and crystallinity, with cortical bone mineral having greater crystallinity (smaller FWHM 002) and crystal orientation (smaller AS_002) for WLb than for RIR hens.

**Table 4:** Summary statistics for bone material properties collected using infrared spectroscopy, thermogravimetry, and X-ray diffraction on tibia cortical and medullary bone for the White Leghorn b and Rhode Island Red populations

Traits	n	Mean ± sem	n	Mean ± sem	n	Mean ± sem	n	Mean ± sem
	Cortical bone		Cortical bone		Medullary bone		Medullary bone	
	White Leghorn		Rhode Island Red		White Leghorn		Rhode Island Red	
*Infrared spectroscopy measurements*								
PO_4_/AmideI	956	3.316 ± 0.009	920	4.232 ± 0.019	961	1.193 ± 0.012	910	1.287 ± 0.017
CO_3_ 1415/AmideI	956	0.951 ± 0.001	919	1.088 ± 0.002	962	0.406 ± 0.002	915	0.426 ± 0.004
MinCO_3_ 1415	958	0.288 ± 0.001	922	0.260 ± 0.001	948	0.356 ± 0.002	920	0.353 ± 0.004
MinCO_3_ 870	958	0.0252 ± 0.0001	920	0.0278 ± 0.0001	948	0.0464 ± 0.0007	914	0.0617 ± 0.0015
*Thermogravimetric measurements*								
H_2_O%	960	11.01 ± 0.04	914	9.57 ± 0.03	941	8.67 ± 0.05	782	8.10 ± 0.05
Organic%	930	23.75 ± 0.03	916	24.51 ± 0.05	962	54.64 ± 0.26	799	57.77 ± 0.39
Mineral%	956	65.24 ± 0.04	915	65.88 ± 0.05	959	36.52 ± 0.24	790	34.01 ± 0.3836
PO_4_%	955	61.81.4 ± 0.04	910	62.67 ± 0.05	962	33.68 ± 0.23	792	31.53 ± 0.36
CO_3_%	958	8.31 ± 0.05	902	3.18 ± 0.02	941	12.59 ± 0.02	780	2.46 ± 0.03
PO_4_/Organic	953	2.607 ± 0.004	910	2.566 ± 0.007	961	0.647 ± 0.007	786	0.596 ± 0.011
CO_3_/Mineral	956	3.42 ± 0.02	901	7.61 ± 0.04	946	2.80 ± 0.03	769	11.92 ± 0.14
*X-ray diffraction measurements*								
FWHM_002 (deg)	954	0.435 ± 0.001	596	0.590 ± 0.002				
FWHM_310 (deg)	955	0.856 ± 0.002	827	1.024 ± 0.003				
AS_002	955	45.3 ± 0.1	885	51.0 ± 0.2				
Oriented fraction	952	0.896 ± 0.004	890	0.514 ± 0.004				

Note only cortical bone was measured for X-ray diffraction

*sem* standard error of the mean

#### Genetic parameters

Only five of the 26 material chemical characteristics of the tibia cortical and medullary bone had significant heritability estimates and showed significant genetic correlation estimates with the mechanical or density properties of the tibia in the WLb and RIR populations (Table 5). Note that the thermogravimetry provided more reliable and quantitative estimates of bone mineral composition than IR spectroscopy in Table 5, for which heritability estimates were not significant.

**Table 5:** Estimates (± SE) of heritability of tibial medullary bone material traits from thermogravimetry and infrared spectroscopy and of genetic correlations with tibia breaking strength and tibia density

Trait in White Leghorn	Trait h^2^	Genetic correlation with tibia breaking strain (h^2^ = 0.27 ± 0.07)	Genetic correlation with tibia density (h^2^ = 0.45 ± 0.08)
PO_4_/AmideI	0.07 ± 0.05	**0.66 ± 0.25**	0.16 ± 0.28
CO_3_1415/AmideI	0.06 ± 0.05	0.64 ± 0.34	**0.98 ± 0.30**
Organic%	**0.14 ± 0.06**	− **0.65 ± 0.22**	− **0.86 ± 0.12**
Mineral%	**0.18 ± 0.07**	**0.61 ± 0.18**	**0.83 ± 0.10**
PO_4_%	**0.21 ± 0.07**	**0.59 ± 0.18**	**0.80 ± 0.10**
PO_4_/Organic	**0.19 ± 0.06**	**0.59 ± 0.19**	**0.85 ± 0.10**
CO_3_/Mineral	0.04 ± 0.04	0.30 ± 0.41	0.61 ± 0.40

Trait in Rhode Island Red	Trait h^2^	Genetic correlation with tibia breaking strain (h^2^ = 0.53 ± 0.08)	Genetic correlation with tibia density (h^2^ = 0.58 ± 0.09)
PO_4_/AmideI	0.09 ± 0.05	**0.68 ± 0.21**	**0.68 ± 0.22**
CO_3_1415/AmideI	0.09 ± 0.05	**0.87 ± 0.21**	**0.77 ± 0.20**
Organic%	**0.41 ± 0.09**	− **0.62 ± 0.11**	− **0.84 ± 0.07**
Mineral %	**0.41 ± 0.09**	**0.62 ± 0.11**	**0.85 ± 0.07**
PO_4_%	**0.39 ± 0.09**	**0.63 ± 0.11**	**0.86 ± 0.07**
PO_4_/Organic	**0.38 ± 0.09**	**0.68 ± 0.10**	**0.90 ± 0.06**
CO_3_/Mineral	**0.12 ± 0.06**	**0.65 ± 0.20**	**0.69 ± 0.18**

Only traits with significant correlations are shown. Data in bold are considered significant. Trait key; (infrared spectroscopy) PO_4_/AmideI, relative amount of mineral to organic matrix in tibia medullary bone; CO_3_1415/AmideI, Total amount of carbonate relative to organic matrix in tibia medullary bone; (thermogravimetry) Mineral%, mineral content in tibia medullary bone; Organic%, organic matter content in tibia medullary bone; PO_4_%, the phosphate content in tibia medullary bone; PO_4_/Organic, the relative phosphate/organic matrix content in tibia medullary bone (thermogravimetry); CO_3_/Mineral, the carbonate to mineral ratio in medullary bone.

In both lines, the preponderance of material chemical factors that showed significant genetic correlations with tibia breaking strength and density concerned the medullary bone and not the cortical bone. In particular, these parameters were related to the amount of mineral in the marrow where the medullary bone is located (i.e., PO_4_/AmideI or PO_4_/Organic) and to bone mineral composition (i.e., Mineral%, Organic%, CO_3_/Mineral) determined by thermogravimetry (Table 5). It should be noted that a number of the IR spectroscopy measurements that relate to mineral content and composition (PO_4_/AmideI and CO_3_1415/AmideI) had heritability estimates that were not significant but they did show moderate genetic correlation estimates with bone strength and density, in line with results for the thermogravimetric measurements (Table 5). The heritability estimates for the thermogravimetric measurements were moderate and ranged from 0.38 ± 0.09 to 0.41 ± 0.09 in the RIR and from 0.14 ± 0.06 to 0.21 ± 0.07 in the WLb hens (Table 5). These measures included Mineral%, PO_4_%, Organic%, PO_4_/Organic. In the RIR hens, these thermogravimetry measurements had high genetic correlation estimates with tibia breaking strength (ranging from 0.62 ± 0.11 to 0.68 ± 0.10) and with tibia bone density (ranging from 0.69 ± 0.18 to 0.90 ± 0.06), while in WLb hens, estimates ranged from 0.59 ± 0.18 to 0.65 ± 0.22 with tibia breaking strength and from 0.80 ± 0.10 to 0.86 ± 0.12 with tibia bone density (Table 5).

Regarding genetic correlations with production parameters such as egg and body mass, and breaking strength, PO_4_/AmideI and CO_3_1415/AmideI had significant negative genetic correlation estimates with egg mass in the RIR population (− 0.59 ± 0.28 and − 0.52 ± 0.21, respectively). Although not significant, because of large standard errors, high genetic correlations were estimated between PO_4_/Organic and late egg production for both the RIR and the WLb populations (0.62 ± 0.43 and 0.81 ± 0.44, respectively).

## Discussion

Our previous results and those presented here show that the potential exists to improve bone quality by selection, since moderate heritability values were found, assuming that a simpler phenotype that does not involve killing hens can be developed for this purpose [24, 26, 33]. The measurements of bone quality used in this study relate to fractures and bone damage, which are traits that the industry aims at improving [33]. We found no evidence for a relationship between post-peak egg production and bone quality, either genetic or phenotypic. However, in line WLb, there was a convincing effect of age at first egg on bone quality. Genetic correlations were also negative between early egg number and cull body mass in the WLb line, which likely reflects the interaction between bone quality and puberty, as puberty onset affects mature body mass [34, 35]. Although the data available for the WLa hens were less detailed, we found no evidence of a genetic correlation between total egg production and tibia breaking strength in this line.

Our results corroborate those reported in [36], where high-producing lines similar to those used here were compared with lines of hens with a similar genetic background but lower egg production. They identified a very weak phenotypic correlation between laying performance and bone quality. However, a number of factors other than egg and shell production were found to differ between the lines in addition to body mass, including AFE, because the more strongly selected lines had started to lay around three-weeks earlier [35]. Overall, as in our study, the authors concluded, that there was limited evidence for a phenotypic association between egg productivity and bone stability traits within the investigated lines [35].

### Age at first egg and bone quality

Age at first egg in poultry is an indication of puberty and, as reported in [37], has a clear genetic determination, which is confirmed by our results for ‘early egg’ number, which is a proxy for age at first egg, bearing in mind that the sign of any correlation is reversed. Age at first egg can be affected by many factors and is often correlated and shares overlapping quantitative trait loci with growth and body mass [35, 38, 39]. If the transition to the physiology associated with egg laying occurs earlier in a hen, several consequences related to that are expected. These include the changes in bone formation, especially the shift to medullary bone formation driven by the requirements for egg shell formation. It is plausible that this might prevent or delay full ossification, especially in the keel, where ossification occurs relatively late [40].

Altering photoperiod and diet during rearing can affect age at first egg [41] but there is conflicting evidence from the literature that this has an effect on bone quality. In a study, in which a 4-day delay in age at first egg was achieved by delaying photostimulation, positive effects on the density and area of medullary and cortical bone in the radius and humerus were observed but bone-breaking strength was not affected [42]. An independent experiment concluded ‘that pullet lighting regimen had little effect on bone mineralization at end of lay’ [43]. Unfortunately, it was not possible to determine whether a difference in age at first egg was achieved in that experiment.

When age at first egg was monitored in an aviary, keel bone fractures, evaluated by palpation, were relatively frequent and were significantly associated with age at first egg, but not with total egg production [44]. This is similar to our results on the WL population. In contrast, another study that investigated bone quality at 70 weeks of age with a wide range of variables, including photoperiodic manipulation that resulted in a 4-day delay in age at first egg, bone quality was better in the earlier maturing hens [45]. No effect of age at first egg on bone breakage at 82 weeks of age was observed [46]. However, it should be noted that the hens were kept in battery cages in the latter two studies, where the probability of breakage is lower [3].

When 13 traditional and 12 commercial breeds were compared for bone quality measured at 55 weeks of age, the traditional breeds had considerably better bone quality and a much later onset of sexual maturity by on average 4 weeks [47]. It was suggested that “…the problems of bone breakage occur in commercial selected layers because they come into lay earlier and continue at higher rates of lay for an extended period of time”. This potentially provides support for both age at first egg and egg production as factors associated with bone quality, although many of the traditional breeds were considerably heavier than the commercial breeds [47]. However, a similar comparison between several laying and broiler breeds concluded that ‘intensive genetic selection for high rates of egg laying has not changed bone size, shape or quality’ [48].

There was no evidence of non-zero genetic correlations of eggshell quality and bone traits with other production traits, except for the significant association between keel bone density and egg breaking strength in the RIR. This suggests that the keel might, in some way, be preferentially used as a depot for calcium for egg shell formation, but one needs to be cautious given the unreliability of the estimate in one line and the absence of significant genetic correlations between egg quality and other bone quality measurements.

### Physico-chemical traits and medullary bone

A second component of this study was to examine the physico-chemical aspects of the cortical and medullary tibia bone and how they relate at the genetic level with the mechanical and density traits for the same bones. Although these data were not collected to demonstrate differences between lines or indeed between medullary and cortical bone types, there are clear differences between the bone types and possibly between lines in IR spectrometry measurements related to mineralisation (PO_4_/AmideI). Similar differences have been observed previously and this confirms that the medullary bone is a relatively labile immature bone type [32]. The observed differences between lines for carbonate content and crystal organisation suggest that the cortical bone mineral is less mature in the RIR hens, which is possibly related to greater remodelling due to the load from their heavier body mass compared to the WL lines [31]. Estimates of heritability were not significant for traits derived from IR spectrometry or X-ray diffraction of cortical bone. IR spectrometry and X-ray diffraction are methods that have been used previously and can detect differences in bone chemistry and structure related to different levels of exercise but not to genetic differences in mechanical properties of laying hen bones [31]. This latter observation agrees with the absence of a genetic component for the variation observed in this study. The exception was the thermogravimetric measurement of tibial medullary bone, which had a significant heritability and was genetically correlated with the different measurements of bone mineralisation. This suggest that better mineralised medullary bone in the tibia correlates with better bone quality. Estimates of genetic correlations between bone quality and medullary bone mineralisation were quite high, especially when one bears in mind that most of the strength of the bone is expected to come from the cortex and not the medullary bone [49]. Although in the case of bone density derived by using X-ray analysis, medullary bone will clearly contribute to the total bone density. In the case of the humerus where the contribution of medullary bone to strength has been studied, there was a positive effect on mechanical properties [50]. This agrees with other results showing the important contribution that medullary bone has on bone mechanical properties [29, 31, 51]. Regardless of the reason for the effect, having a well-mineralised medullary bone is likely an important genetic factor in determining the quality of the skeleton towards the later stages of egg production. Heritability was estimated in one study and it was found to be relatively low (0.13) [33], perhaps suggesting that a better phenotype is needed to estimate its quality. While it is possible that the contribution of the medullary bone to the strength and quality of a bone is direct, it is also possible that well mineralised medullary bone protects the cortical bone indirectly against resorption by ensuring an adequate supply of calcium during eggshell formation cycle, thus preventing osteoclast breakdown of the cortical bone. Interestingly, we found a high positive, albeit non-significant, genetic correlation between late egg production and mineralisation of the medullary bone in both lines, which suggests that mineralisation of the medullary bone was higher in hens laying more eggs over the main part of lay, although this clearly needs confirmation. The only significant genetic correlations between material properties and production traits were those of PO_4_/AmideI and CO_3_1415/AmideI with egg mass in the RIR population. However, these material property traits had low heritability estimates. The genetic correlation estimates were negative, which indicates that lower mineralisation in the medullary bone may be associated with larger eggs.

### How can bone quality in laying hens be improved in light of these studies?

The methods that we described here to assess bone quality require *post-mortem* analysis and are relatively time-consuming for use in genetic selection. Studies to find suitable methods for measuring bone quality on live animals on a sufficiently large scale are ongoing and simple methods such as keel bone palpation may be useful in this regard [23]. Methods that have been tested in other studies [52] may be worth revisiting, as technology has improved. It may also be feasible to adapt some of the new methods to estimate medullary bone mineralization based on X-ray, which could be a potential goal for selection and for management strategies, alongside keeping attention on age at first egg in breeding programmes. These strategies, combined with management, improved housing, and optimal nutrition have the potential to improve bone quality and welfare [9].

## Conclusions

This study has provided some clear priorities in terms of managing laying hens and targeting research for the future. First, puberty onset (age at first egg) should be examined closely and care should be taken to ensure that adequate bone development occurs before the hens enter lay. Manipulation of puberty onset may offer scope to improve overall bone health in layers. Second, it appears that having a well-mineralised medullary bone is important in protecting the overall quality of the skeleton of laying hens. The development and maintenance of adequate medullary bone and potentially the genetic factors that promote its deposition, may confer important advantages when it comes to improving bone quality. Lastly, assuming selection is practiced carefully and methods are available, improving bone quality should not necessarily result in lower egg production or egg quality. This agrees with previous results, where selection for improved bone quality did not result in poorer egg production. It also suggests that the move towards longer laying periods will not necessarily adversely affect bone quality, but this will need to be carefully monitored.

## Data Availability

The datasets analyzed during the current study containing pedigree information are not publicly available due to commercial sensitivity. The data has been archived with the following doi; https://doi.org/10.7488/6f5c32dc-da11-477a-9ded-6f31fb10821e.
